# Traffic Flow Prediction and Analysis in Smart Cities Based on the WND-LSTM Model

**DOI:** 10.1155/2022/7079045

**Published:** 2022-08-02

**Authors:** SuYuan Ma, MingYe Zhao

**Affiliations:** ^1^School of Marxism, Guangzhou University of Chinese Medicine, Guangzhou 510006, China; ^2^Business School of International Medicine, China Pharmaceutical University, Nanjing 210009, China

## Abstract

Aiming at the problem that the road traffic flow in intelligent city is unevenly distributed in time and space, difficult to predict, and prone to traffic congestion, combined with pattern recognition and big data mining technology, this paper proposes a research method to analyze and mine the daily travel patterns of urban vehicles. This paper proposes a WND-LSTM model, which mainly includes data preprocessing, data modelling, and model implementation, to analyze the similarity of travel patterns in seasonal changes. Combining the data mining results with the data mining results, the daily travel model of road traffic vehicles in intelligent city is established. The results of the case study showed that the WND-LSTM model outperformed ARIMA (88.48%), LR (65.79%), SVR (70.46%), KNN (68.21%), SAEs (66.95%), GRU (68.43%), and LSTM (70.41%) in MAPE, respectively, with an average accuracy improvement of 71.25% (MAPE of 0.651%).

## 1. Introduction

At present, with the rapid development of the global economy, the quality of life in a country is booming, especially the rapid changes in clothing, food, housing, and transportation, and the number of household cars is increasing year by year [1]. The urban motor vehicle ownership rate is growing at a rate of no less than 11.5% per year [2]. Despite the rapid development planning of cities and towns in China, the construction of transportation facilities is far from catching up with the rapid growth of vehicles. With the continuous popularization of people's means of transportation, the problems of traffic congestion and traffic pollution are becoming more and more serious [3]. The traditional urban transportation system is facing severe challenges because it seriously restricts the quality of life of citizens.

Consistent with today's urban traffic planning ability, how to optimize the road network capacity and improve the decision-making ability has always been the most difficult problem in traffic management [4]. As one of the core contents of smart city, its goal is to create a modern, efficient, and stable traffic management system by using the technology of cutting-edge fields (computer information, automation, and artificial intelligence) so as to maximize the road capacity, alleviate or even maximize the road capacity, alleviate or even solve the problem of traffic congestion, and make all citizens live in a stable and orderly environment [5]. As the core element of intelligent transportation system (ITS), the research on transportation network operation mode and people's daily travel behavior can provide real and reliable data information for decision-makers so as to effectively deploy transportation network resources [6].

Today, we are in the age of big data. Data has permeated every industry and every function and has become an important factor in production [7]. As a fundamental part of ITS, the accuracy of traffic flow information collection is particularly important. A wide range of traffic flow information collection technologies such as laser sensing, GPS positioning information microwave detection, geomagnetic coil detection, and camera recording are widely used in this field, and these sensors can be found almost everywhere, generating huge amounts of real-time traffic data information all the time, with information volumes of up to petabytes [8]. The question of how to fully exploit the “gold” of this data has been met with great enthusiasm by a wide range of knowledgeable people [9]. Big data mining method can use high-speed computer cluster or server cluster to process massive original traffic video data, carry out information mining, extract valuable rule information in a short time, and realize the knowledge transformation of data. Big data technology combines high-speed computing power and powerful storage capacity and will play a key role in reasonably allocating traffic network resources and avoiding various traffic problems [10].

Image recognition and big data mining are widely used artificial intelligence technologies. At present, they are playing a great role in promoting a wide range of industries, including government functions, medical care, industry, commerce, and military. However, the author found that their application in the field of transportation still has great potential. Based on the above background, this paper aims to combine a new perspective with the road network, use the massive traffic data generated by the well-equipped monitoring system in the traffic road network, and use the Hadoop big data processing framework, combined with *K*-means clustering algorithm and Δ Apriori association algorithm, from the perspective of data feature selection, obtains license plate information through image recognition, mines traffic flow model, and transforms big data into small data. Data are selected from the perspective of data characteristics, big data are converted into small data, and traffic network operation model is established [1]. This allows more targeted data and significantly reduces redundant data, so it has the advantages of cost-effectiveness and efficiency. Based on the traffic flow model and the accurate space-time mode of each vehicle, the designed route guidance algorithm provides real-time and effective route planning services for end users, allows each vehicle to find the route that best meets its requirements at the lowest user cost, reduces the concentrated load on the road, and reduces the causes of traffic congestion to a certain extent. This reduces the causes of traffic congestion, reduces the pressure of traffic management departments, and saves the waste of human and material resources [11].

## 2. Related Work

Since the turn of the century, computer information technology has developed rapidly, and the era of big data has arrived. Once the concept of big data mining methods was introduced, it has contributed to huge changes in a large number of traditional industries, such as energy, health care, and services, and has promoted the development of many industries. Many experts and scholars are also using the current hot big data analysis methods to solve transportation problems. Reference [12] modelled the public transport system based on discrete choice in terms of the impact of comfort and other aspects on passengers' choice of travel mode, and [13] modelled the urban public transport system based on Neighborhood search algorithm. A group intelligence model-based approach is proposed in [14] for open-ended route planning. Deletion algorithm and Ms algorithm are proposed for the shortest path problem, respectively, by [15]. In [16], a model for rail transit path selection is developed based on the Logit model. The model clustering analysis is applied in [17] for efficient detection of the forward speed of the traffic within the detection range. In [18], it is proposed to combine road intersection vehicle data, traffic facility data and traffic accident data with the help of establishing a GIS platform for accident analysis and detection. Reference [19] designed an Android-based GIS online navigation system. In [14], in order to solve the parameter convergence problem, weights' calculation is added to the particle swarm algorithm, and the right of way is dynamically adjusted according to the roadway load change by combining the discrete selection of fuzzy rules. Reference [15] ensembles neural networks and Bayesian methods for predicting short-time traffic. Reference [16] applies multiprediction models to the field of road section load research. Reference [17] proposed a dynamic stochastic shortest path optimisation method to provide guidance on vehicle path optimisation through stochastic meritocratic planning.

## 3. WND-LSTM Model

### 3.1. Model Overview

**Figure 1:**
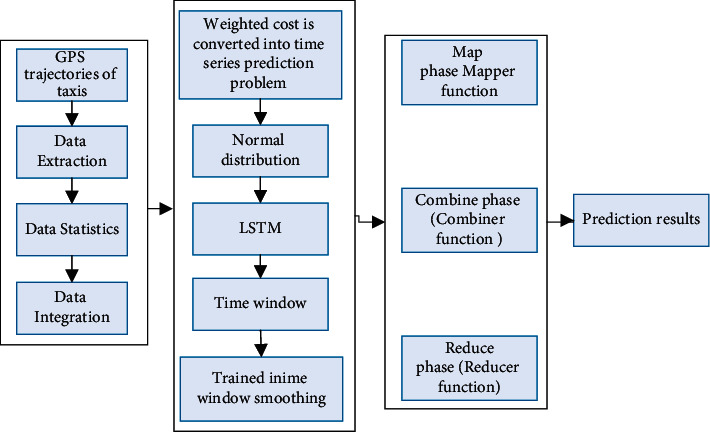
WND-LSTM model framework.

As shown in Figure 1, our proposed WND-LSTM model mainly consists of three parts: data preprocessing, data modelling, and model implementation. Firstly, in the data preprocessing, the MapReduce parallel processing framework is used to process large-scale taxi GPS track data on the Hadoop distributed computing platform to achieve data extraction, data statistics, and data integration; secondly, the distributed WND-LSTM model is built on Hadoop, and the LSTM model is weighted with time windows and normal distributions. Finally, the WND-LSTM model was executed on MapReduce using Mapper, Combiner, and Reducer functions to improve the efficiency and scalability of the model predictions.

In addition, to address the storage and computational issues that exist when using independent learning models to handle large data of traffic, we introduce a generic MapReduce framework distributed modelling architecture for traffic flow prediction (MF-TFF) in the proposed WND-LSTM model [18].

### 3.2. Data Preprocessing

**Figure 2:**
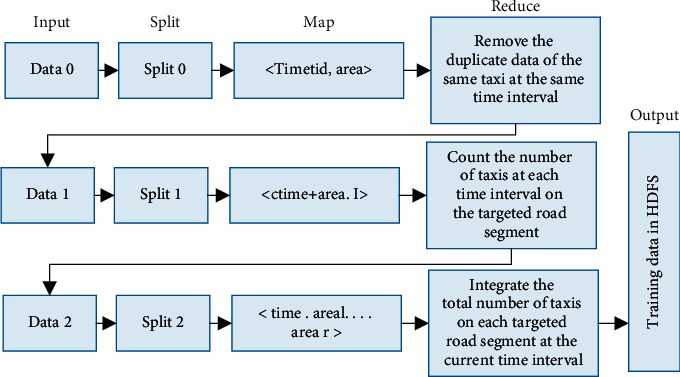
MapReduce-based data preprocessing process.

As the taxi GPS track data is not only spatiotemporally correlated and nonlinear, but also has the characteristics of huge data volume, variety, high value, low density, and fast speed, it is necessary to do data preprocessing work on the original GPS track data. In this paper, we extracted taxi GPS track point record data from the target road section for data preprocessing respectively. The specific process is shown in Figure 2. The data preprocessing process on MapReduce consists of three main tasks.

#### 3.2.1. Task 1: Data Extraction

Extract the taxi vehicles present in the area of interest on the same day via GPS. Define a key-value pair < key1, value1> in the first Map task, where key1 represents the time and vehicle ID and value1 represents the number of the area location. Input to the Reduce task, in the Reduce stage, by sorting the time and vehicle number, remove the duplicate data of the same vehicle at the current time, and write to HDFS; see Algorithm 1 for details.

#### 3.2.2. Task 2.Data Statistics

Count the number of taxis in each road section every five minutes, read in the information saved in the previous task in the second Map task, and define the key values. In the second Map task, the information saved in the previous task is read in and the key-value pair < key2, value2 > is defined, with key2 representing the time and area number and value2 being entered as count 1. The database may be missing data for the current time point due to the absence of vehicle information at a certain time point in the previous task. Therefore, a key-value pair < keys, values > needs to be added to the database at this point in time as a marker to ensure that the number of taxis at this point in time is not missing. As a marker for this moment in time, ensuring that each day has a fixed point in time.

#### 3.2.3. Task 3: Data Integration

In the third Map task, the number of taxis counted in the second task is read in, and the key-value pair < key3, value3> is defined, where the time point is used as key3 and value3 represents the number of taxis counted in each road section.

### 3.3. Model Construction

The LSTM model has a unique structure that not only inherits the general characteristics of RNNs, but also solves the problem of gradient explosion and gradient disappearance when the values are too large or too small. The structure of the LSTM consists of an input layer, an implicit layer, and an output layer, which also contains a memory cell. The LSTM is structured as an input layer, an implicit layer, an output layer, a memory cell, and three gating units: an input gate, an output gate, and a forgetting gate, which are used to control the flow of information in the input and memory cells, allowing the LSTM to better remember useful information and handle long-term time-dependent relationships.

In an LSTM network, assuming an input *x*=(*x*_1_, *x*_2_,…, *x*_*t*_) time series, a time series of *h*=(*h*_1_, *h*_2_,…, *h*_*t*_) in the implicit layer and a time series of *y*=(*y*_1_, *y*_2_,…, *y*_*t*_) in the output layer, the calculation can be performed by the following equation:

(1) $$\begin{array}{c} y=W_{hy}h_{y}+b_{y},\\ h_{t}=H\left(W_{xh}x_{t}+W_{hh}h_{h-1}+b_{h}\right), \end{array}$$

where the implicit layer function of the LSTM can be obtained by the following equation:

(2) $$\begin{array}{c} i_{t}=\sigma \left(W_{xi}x_{t}+W_{hi}h_{t-1}+W_{ci}c_{t-1}+b_{i}\right),\\ f_{t}=\sigma \left(W_{xf}x_{t}+W_{hf}h_{t-1}+W_{cf}c_{t-1}+b_{f}\right),\\ c_{t}=f_{t}c_{t-1}+i_{t}\text{g}\left(W_{xc}x_{t}+W_{hc}h_{t-1}+b_{c}\right),\\ o_{t}=\sigma \left(W_{xo}x_{t}+W_{ho}h_{t-1}+W_{co}c_{t-1}+b_{o}\right),\\ h_{t}=o_{t}h\left(c_{t}\right), \end{array}$$

where *i*, *o*, *f*, and *c* represent the input gate, output gate, forgetting gate, and memory cell, respectively, *W* is the weight matrix, *b* is the bias vector, and *σ* is the activation function.

In this work, as a result of simply using the traffic flow sequences upstream and downstream and at each bifurcation intersection to participate in the training, which does not consider the impact of each intersection on the current roadway in the time axis, thus proposing a relatively novel weighting method. A relatively novel weighting method is proposed to improve the prediction accuracy of short-time traffic flow. The LSTM is weighted to find the cost by using the characteristics of normal distribution and time window smoothing, and the cost is transformed into a time series. The problem of predicting traffic flow in a series of single variables over time is used to predict the state of the variables at the current moment.

Therefore, the MapReduce parallel computing framework on Hadoop optimises the traditional LSTM neural network. Firstly, the LSTM neural network is weighted to find the cost using a time window and normal distribution, and the cost is used as a time series prediction problem to form a time-dependent single variable series to predict the state of the variable at the current moment; secondly, on the Hadoop distributed computing platform with large data of taxi trajectories, we can obtain the traffic with time series by the above data preprocessing method. Finally, based on the obtained traffic sequence dataset, we select the target road section and then use WND-LSTM to build a traffic flow prediction model, which consists of the following three steps.

**Step 1 .**

The horizontal intersection section is considered as a network diagram, and *V*_*t*_^*i*^ is defined as the traffic flow value collected from the target section at time *t*. Using the traffic flow sequence at time *t* to predict the traffic flow at time *t*+1 *V*_*t*+1_^*i*^, with a sampling interval of 5 minutes, the traffic flow sequence can be expressed as *O*_*i*_={*V*_1_^*i*^, *V*_2_^*i*^,…, *V*_*n*_^*i*^}*t*, where *i* = 1, 2, *R* (*n* = 288), and the predicted traffic flow of any target road is predicted by the current moment of the road section and the related intersection, using the LSTM recurrent neural network. If section *i* junction is used as the predicted junction, the prediction model inputs and outputs are

(3) $$\begin{array}{c} X=\left(O_{1},O_{2},\ldots ,O_{R}\right),\\ Y=\left(O_{t+l}^{i}\right). \end{array}$$

**Step 2 .**

A normal distribution is one in which the random variable *x* follows a normal distribution with a mathematical expectation of *μ* and a variance of *σ*^2^, denoted as *N*(*μ*, *σ*^2^). The probability density function is the expectation of the normal distribution *μ* that determines its location (centre line) and its standard deviation *σ* that determines the magnitude of the distribution. Since it is clearly not reasonable to simply use the traffic flow sequences upstream and downstream and at each bifurcation to participate in the training and does not take into account the influence of each intersection on the current roadway in the time axis, the WND-LSTM uses the idea of a normal distribution to weight the cost of each road segment and then uses the cost as a time series prediction problem to form a single variable sequence over time to predict the state of the variable at the current moment, which allows for artificial control purposes. The mean value of the normal distribution is the target road section, where the empirical value is 0.6 (which can be any reasonable value) and *x* is the discrete value of the traffic volume on each road section.

(4) $$\begin{array}{c} f\left(x\right)=\frac{1}{\sqrt{2\pi \delta }}\text{exp}\left(-\frac{{\left(x-\mu \right)}^{2}}{2\delta^{2}}\right). \end{array}$$

**Step 3 .**

Based on the above steps, the traffic flow on the target roadway at time *t* of the day is selected as *V*_*t*_^*i*^, and *V*_*t*−1_^i^, *V*_*t*−2_^i^, *V*_*t*−3_^i^ of the current historical moment is taken by window smoothing, and a window of size 4 is formed and fed into the training, in the following form:

(5) $$\begin{array}{c} V=\left[\begin{array}{c} V_{1}\\ \;V_{2}\\ \vdots \\ V_{n-3} \end{array}\right]=\left[\begin{array}{cccc} v_{1} & v_{2} & v_{3} & v_{4}\\ v_{2} & v_{3} & v_{4} & v_{5}\\ \vdots & \vdots & \vdots & \vdots \\ v_{n-3} & v_{n-2} & v_{n-1} & v_{n} \end{array}\right]. \end{array}$$

## 4. Experimental Results and Analysis

### 4.1. Experimental Environment

The experimental environment was built on a Hadoop cluster with a MapReduce framework and ArcGIS platform, where the base hardware was an HP Z400 workstation with an Intel Xeon 3500 CPU and ECC and DDR3 8.0 GB RAM. We ran all experiments with Hadoop 3.1.1 using Java, Python on Ubuntu 18.64 OS [20, 21].

### 4.2. Experimental Data

We used real taxi GPS trajectory data (approximately 25 GB) as the experimental data for this case study, which was produced from November 5 to November 18, 2021, in Beijing via 12,000 taxis.

**Figure 3:**
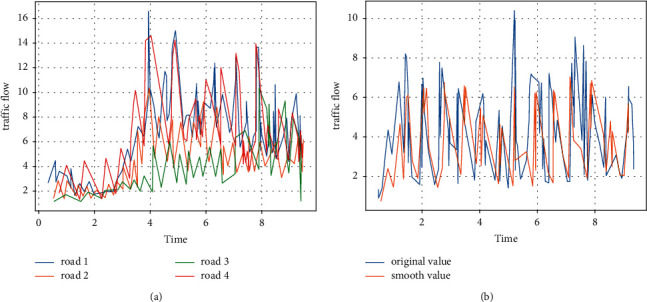
Experimental data smoothing. (a) Traffic flow on 4 road sections. (b) Kalman filter smoothing.

These data consisted of 968 million GPS trajectory points, forming a visible traffic network map of Beijing, China, as shown in Figure 3(a).

In this experiment, we selected datasets from the above datasets for weekdays (November 5 to November 18, 2012) and also divided the datasets into five groups according to 1 day, 3 days, 6 days, 10 days, and 14 days in order to verify scalability, where 168 data were selected as the test set and the rest as the training set at 1 day, and 1 day was selected as the test set and the rest as the training set for all 3 days, 6 days, 10 days, and 14 days [22].

In particular, using the method described in the text, the above dataset was preprocessed based on MapReduce, and the traffic flows of the four road sections are shown in Figure 3(a). In addition, to make the experimental data valid, we used KF filtering to smoothen the set of the counted data and normalised the data to between [0, 1] by the MinMaxScaler function, as shown in Figure 3(b).

### 4.3. Performance Assessment

In general, the more hidden layers a neural network has, the deeper the model is, and the better the representation and learning ability of the model, but it also leads to gradient loss and increases the difficulty of training. In this experiment, the model is trained on a limited dataset. The first part is an input layer of dimension 11, the second part is an LSTM hidden layer of dimension 64, and the third part is an output 1 vector, with batch taken as 1 (grid search) and 100 iterations using a sigmoid optimiser [23].

To evaluate the prediction accuracy of the proposed WND-LSTM model in terms of time windows and normal distribution, we compared it with LSTM, W-LSTM (LSTM with time window smoothing), and ND-LSTM (LSTM with normal distribution).

**Figure 4:**
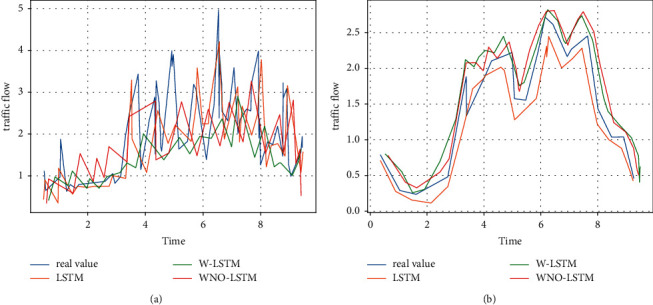
LSTM, W-LSTM, ND-LSTM, and WND-LSTM smoothing treatments.

**Figure 5:**
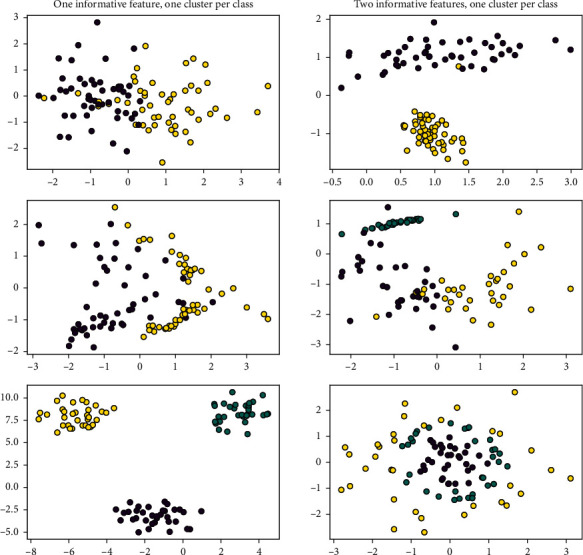
LSTM, W-LSTM, ND-LSTM, and WND-LSTM evaluation metrics.

With the same experimental dataset, we used KF for data smoothing based on similar traffic flow characteristics and, in particular, for experimental results with the same trend, we chose a dataset of 14 days due to spatial constraints [24], as shown in Figure 4. More importantly, we compared the MAPE values of LSTM, W-LSTM, ND-LSTM, and WND-LSTM, presenting the values of MOEs (MAPE, MAE, RMSE, and ME) for LSTM, W-LSTM, ND-LSTM, and WND-LSTM, respectively, as shown in Table 1 and Figure 5.

Table 1 reports the MOEs for the five datasets of the four LSTM methods, and it can be seen that the MAPE of the WND-LSTM method is lower than that of the other methods. The MAPEs of the WND-LSTM model are significantly lower than those of the other models in most cases, especially for the 14-day dataset, where the MAPEs of the WND-LSTM are lower than those of the LSTM, W-LSTM, and ND-LSTM by 70.41%, 68.92%, and 46.77%, respectively. In particular, the MAPE value for WND-LSTM is 0.651%, indicating a high predictive power [20].

**Figure 6:**
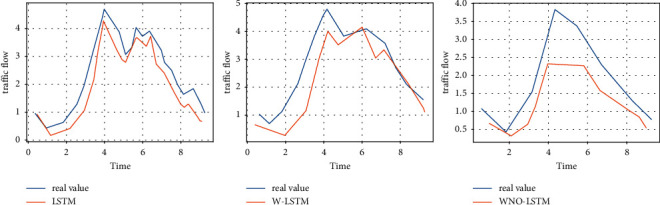
Comparison of prediction results of different models on the same dataset.

**Figure 7:**
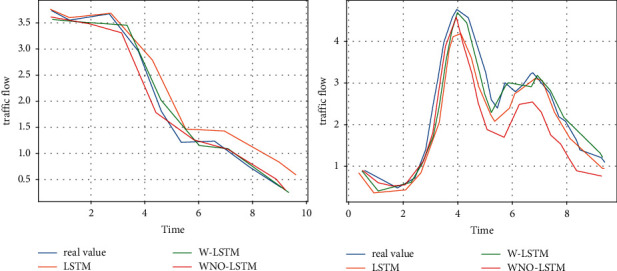
Prediction results of LSTM, W-LSTM, and WND-LSTM models for different datasets.

Also for a more visual illustration, Figure 6 presents the traffic flow prediction results produced by WND-LSTM compared to other LSTM models (given the similarity of trends in each set of experimental results, a 10-day dataset was chosen to present the results). As the dataset increases, the accuracy of the WND-LSTM model increases and the timeliness becomes better, as shown in Figure 7.

To further validate the accuracy of the WND-LSTM model, we compared it with advanced differential autoregressive moving average (ARIMA), logistic regression models (LR), support vector regression (SVR), *K*-nearest neighbour (KNN), stacked autocoding neural networks (SAEs), gated recurrent neural units (GRUs), and long- and short-term memory neural network (LSTM) prediction models, and the experimental results are shown in Table 2.

## 5. Conclusions

In this paper, we propose a distributed LSTM weighting model, WND-LSTM, which combines time windows and normal distribution to improve the accuracy of short-time traffic flow prediction (TFP). In addition, we implement the proposed WND-LSTM model on a MapReduce parallel processing framework to address the scalability and efficiency of short-time traffic flow prediction and use KF to filter the raw GPS taxi movement trajectory data to remove anomalies for discrete data smoothing. The results of the case study showed that the WND-LSTM model outperformed ARIMA (88.48%), LR (65.79%), SVR (70.46%), KNN (68.21%), SAEs (66.95%), GRU (68.43%), and LSTM (70.41%) in MAPE, respectively, with an average accuracy improvement of 71.25% (MAPE of 0.651%).

## Figures and Tables

**Table 1:** Comparison of evaluation metrics for LSTM, W-LSTM, ND-LSTM, and WND-LSTM models.

Days		MOSEs		Models	
		ND-LSTM	W-LSTM	LSTM	WND-LSTM
1 day	MAPE	6.324	3.195	6.122	2.159
	MAE	0.108	0.055	0.137	0.056
	RMSE	0.147	0.066	0.184	0.372
	ME	0.374	0.131	0.368	0.372

3 days	MAPE	5.579	2.973	5.587	1.793
	MAE	0.144	0.081	0.145	0.053
	RMSE	0.202	0.146	0.275	0.102
	ME	0.612	0.581	1.072	0.412

6 days	MAPE	1.268	1.894	2.756	0.194
	MAE	0.015	0.029	0.044	0.012
	RMSE	0.018	0.036	0.059	0.015
	ME	0.041	0.109	0.164	0.039

10 days	MAPE	4.305	1.931	2.988	0.715
	MAE	0.056	0.035	0.045	0.015
	RMSE	0.064	0.004	0.054	0.018
	ME	0.121	0.135	0.141	0.048

14 days	MAPE	1.223	2.095	2.200	0.651
	MAE	0.016	0.032	0.03	0.014
	RMSE	0.02	0.042	0.037	0.014
	ME	0.045	0.114	0.105	0.044

**Table 2:** Comparison of evaluation metrics between WND-LSTM and other advanced models.

Days	MOEs	Models							
		ARIMA	LR	SVR	KNN	SAEs	GRU	LSTM	WND-LSTM
1 day	MAPE	5.297	2.558	3.493	2.458	4.473	4.634	6.122	2.159
	MAE	0.095	0.04	0.062	0.047	0.078	0.076	0.137	0.056
	RMSE	0.123	0.005	0.08	0.066	0.084	0.092	0.184	0.096
	ME	0.302	0.116	0.163	0.245	0.154	0.219	0.369	0.372

3 days	MAPE	6.031	2.819	6.701	4.297	2.308	4.656	2.587	1.795
	MAE	0.138	0.067	0.273	0.16	0.049	0.131	0.145	0.053
	RMSE	0.196	0.096	0.764	0.4	0.069	0.25	0.275	0.102
	ME	0.689	0.438	3.1	1.629	0.269	0.972	1.722	0.410

6 days	MAPE	3.835	1.946	2.466	2.037	2.205	1.988	2.756	0.75
	MAE	0.056	0.030	0.034	0.031	0.032	0.032	0.044	0.012
	RMSE	0.072	0.036	0.041	0.038	0.038	0.042	0.059	0.015
	ME	0.182	0.086	0.095	0.094	0.087	0.114	0.164	0.039

10 days	MAPE	5.842	1.952	2.509	2.061	2.515	2.016	2.988	0.716
	MAE	0.112	0.038	0.042	0.04	0.052	0.039	0.045	0.015
	RMSE	0.139	0.047	0.05	0.05	0.066	0.052	0.054	0.018
	ME	0.42	0.144	0.146	0.167	0.155	0.166	0.141	0.048

14 days	MAPE	5.652	1.903	2.204	2.046	1.97	2.065	2.2	0.51
	MAE	0.084	0.028	0.03	0.03	0.029	0.031	0.03	0.01
	RMSE	0.106	0.036	0.038	0.04	0.037	0.041	0.037	0.014
	ME	0.298	0.1	0.113	0.12	0.103	0.106	0.105	0.044

## Data Availability

The experimental data used to support the findings of this study are available from the corresponding author upon request.
